# PKB-Mediated Thr^649^ Phosphorylation of AS160/TBC1D4 Regulates the R-Wave Amplitude in the Heart

**DOI:** 10.1371/journal.pone.0124491

**Published:** 2015-04-29

**Authors:** Chao Quan, Bingxian Xie, Hong Yu Wang, Shuai Chen

**Affiliations:** MOE Key Laboratory of Model Animal for Disease Study, Model Animal Research Center, Nanjing Biomedical Research Institute, Nanjing University, Nanjing, 210061, China; University of Cincinnati, College of Medicine, UNITED STATES

## Abstract

The Rab GTPase activating protein (RabGAP), AS160/TBC1D4, is an important substrate of protein kinase B (PKB), and regulates insulin-stimulated trafficking of glucose transporter 4. Besides, AS160/TBC1D4 has also been shown to regulate trafficking of many other membrane proteins including FA translocase/CD36 in cardiomyocytes. However, it is not clear whether it plays any role in regulating heart functions *in vivo*. Here, we found that PKB-mediated phosphorylation of Thr^649^ on AS160/TBC1D4 represented one of the major PAS-binding signals in the heart in response to insulin. Mutation of Thr^649^ to a non-phosphorylatable alanine increased the R-wave amplitude in the AS160^Thr649Ala^ knockin mice. However, this knockin mutation did not affect the heart functions under both normal and infarct conditions. Interestingly, myocardial infarction induced the expression of a related RabGAP, TBC1D1, in the infarct zone as well as in the border zone. Together, these data show that the Thr^649^ phosphorylation of AS160/TBC1D4 plays an important role in the heart’s electrical conduction system through regulating the R-wave amplitude.

## Introduction

Insulin signaling in the heart plays a critical role in regulating cardiac development, postnatal growth, metabolisms and cardiac contractility [1]. Insulin resistance under pathological conditions such as type II diabetes is a risk factor for development of heart failure after myocardial infarction [2]. In the past few decades, genetically-modified mouse models have helped to elucidate the roles of myocardial insulin signaling under physiological conditions, and the contributions of its impairment in the adaptation to myocardial infarction [3–6]. Cardiomyocyte-specific knockout of insulin receptor (IR) in mouse reduces cardiomyocyte size and moderately impairs cardiac performance [3]. The mild decrease in cardiac performance in this mouse model is due to the compensation of insulin-like growth factor 1 receptor (IGF1R) in cardiomyocytes. When both IR and IGF1R are deleted in cardiomyocytes, the double knockout mouse develops dilated cardiomyopathy and eventually dies of heart failure [7]. Phosphoinositide-dependent protein kinase 1 (PDK1), an important protein kinase downstream of both receptor kinases, mediates insulin- or IGF1-stimulated activation of protein kinase B (PKB, also known as Akt). Likewise, deletion of PDK1 in cardiac muscle results in a decrease in cardiomyocyte size, and the mouse develops cardiomyopathy and dies of heart failure [6]. While cardiac-specific knockout PKBβ/Akt2 impairs insulin-stimulated glucose uptake into cardiomyocytes [5], deletion of PKBα/Akt1 results in heart developmental defects and decreased heart function, which can be partially rescued by downregulation of p38α [8, 9]. These studies highlight the importance of insulin-PKB/Akt pathway in regulating heart functions, and its deregulation directly links to the pathogenesis of cardiac diseases. However, factors downstream of PKB/Akt mediating the pathogenesis of these heart diseases are still not very clear.

AS160/TBC1D4 is a Rab GTPase activating protein (RabGAP) that was initially identified as a phosphorylated PKB substrate in adipocytes [10]. A related RabGAP, TBC1D1, can also be phosphorylated by PKB [11, 12]. Both AS160/TBC1D4 and TBC1D1 regulate translocation of glucose transporter 4 (GLUT4) storage vesicles [10, 12]. In addition, AS160/TBC1D4 has been implicated in regulating intracellular trafficking of FA translocase/CD36 [13], aquaporin-2 [14], epithelial sodium channel [15], and Na^+^/K^+^-ATPase [16]. Thr^649^ on AS160/TBC1D4 (numbering according to the mouse protein) is a critical PKB phosphorylation site that binds to the 14-3-3 regulatory proteins [17, 18]. A knockin mouse in which Thr^649^ on AS160/TBC1D4 is mutated to a non-phosphorylatable alanine develops insulin resistance in skeletal muscle and exhibits an increased ratio of heart to body weight [19].

In this study we investigated the impacts of the AS160-Thr^649^ phosphorylation on the heart’s electrical conduction system and heart functions under normal and myocardial infarction conditions using the AS160^Thr649Ala^ knockin mouse model.

## Materials and Methods

### Materials

Recombinant human insulin was from Novo Nordisk (Bagsvaerd, Denmark), microcystin-LR was from Taiwan Algal Science Inc (Taoyuan, Taiwan, China), and Protein G-Sepharose was from GE Healthcare (Little Chalfont, Buckinghamshire, UK). All other chemicals were from Sigma-Aldrich (St. Louis, Missouri, USA) or Sangon Biotech (Shanghai, China).

### Antibodies

The sheep antibody against phosphorylated Ser^231^ on TBC1D1 was previously reported [11]. The flotillin-1 antibody (Cat No. sc-25506) was from Santa Cruz (Dallas, Texas, USA). The antibodies that recognise phosphorylated Ser^473^ on PKB (Cat No. 9271), total PKB (Cat No. 9272), and total TBC1D1 (Cat No. 4629), and the phospho-Akt substrate (PAS) antibody (Cat No. 9611) were from Cell Signaling Technology. The total AS160/TBC1D4 (Cat No. 07–741) was from Millipore. The antibody that recognises phosphorylated Thr^649^ on AS160/TBC1D4 (Cat No. 441071G) was from Life Technologies. The GAPDH antibody (Cat No. G8795) was from Sigma, and the cTnT antibody (Cat No. MA5-12960) was from Thermo Scientific.

### Mouse husbandry and insulin injection

The Ethics Committee at Model Animal Research Center of Nanjing University approved the mouse procedures used in this study. Mice were maintained under a light/dark cycle of 12 h, and had free access to food and water unless stated. The AS160^Thr649Ala^ knockin mice are as previously described [19, 20]. In total, 90 mice were used in this study.

Intraperitoneal injection of insulin (150 mU insulin per g of body weight) was carried out in anaesthetized mice that had been restricted from food access overnight (16 hours). After insulin injection, mice were kept on a heating pad for 20 min before cervically dislocated. Hearts were immediately removed and snap-frozen in liquid nitrogen.

### Heart protein extraction and western blot

Mouse hearts were homogenized in lysis buffer using a Polytron homogenizer (Kinematica, Luzern, Switzerland), and lysed on ice for 30 min as previously described [19]. Protein extracts were obtained after tissue debris was removed via centrifugation. Proteins were electrophoretically separated via SDS-PAGE, and immunoblotted onto nitrocellulose membranes that were subsequently incubated with primary antibodies. Membranes were further incubated with horseradish-peroxidase-conjugated secondary antibodies. Chemiluminescence signals were detected using a gel documentation system (Syngene, UK) after ECL substrates were added (GE Healthcare, UK).

### Electrocardiography (ECG)

Mice were anaesthetized with 2% isoflurane mixed with 100% O_2_ (0.5 L/min), and anesthetized animals were maintained by continuous gaseous isoflurance (1% to 1.5%) mixed with 100% O_2_ (0.5 L/min). The electrocardiograms were collected with PowerLab 8/30 (AD, ML870) and dual bio amp/stimulator (AD, ML408) system as previously described [21]. The parameters were measured by using the ECG analysis module of labchart software.

### Echocardiography (Echo)

Mice were anaesthetized with gaseous isoflurane as afore-mentioned. Echocardiography was performed on anaesthetized mice using a Vevo 770 high-resolution *in vivo* micro-imaging system (VisualSonics, inc) with a 30MHz RMV-707B ultrasonic probe as previously described [21]. Briefly, mice were shaved on their chests, and then placed on a heating pad in a supine position with their chests covered with ultrasound transmission gel. The ultrasonic probe was immobilized with a 90° angle between the probe and the heart to collect M-mode pictures. The following parameters were measured on the M-mode tracing and averaged from 6 cardiac cycles: left ventricle anterior wall (LVAW), left ventricle posterior wall (LVPW), left ventricle internal dimension (LVID) of systole and diastole. The calculation of ejection fraction (EF) formula is EF% = [(LV Vol;d—LV Vol;s)] x 100%, and fraction shortening (FS) is calculated as FS% = [(LVID;d—LVID;s)/LVID;d] x 100%.

### Induction of myocardial infarction

Mice were anaesthetized via intraperitoneal injection of a mixture of ketamine (100 mg per kg of body weight) and xylazine (10 mg per kg of body weight). During thoracotomy, mice were incubated endotracheally and ventilated using a Harvard Mouse Mini-Vent (type 845, Havard Apparatus, Germany) (stroke volume: 0.3 mL; rate: 120 times/min). Thoracotomy on the left side of the chest was performed via the fourth intercostal space to provide access to the beating heart. The pericardium was opened, and the left anterior descending coronary artery (LAD) was ligated with silk suture near its origin between the pulmonary outflow tract and the edge of the left atrium. After artery ligation, the chest was closed, and mice were kept on the heating pad until they became conscious again. The control mice with sham operation underwent the same procedure of thoracotomy and pericardium opening but with no ligation of LAD. After surgery, mice were monitored daily and administered with carprofen if they experienced distress. None of the mice died due to surgery.

### Masson’s staining

Hearts were isolated immediately after termination of mice and fixed in 4% PFA overnight at 4°C. Hearts were embedded in paraffin and cut into 5-μm-thick slices for Masson’s staining. Briefly, after deparaffinization and rehydration, the sections were first stained with Biebrich scarlet for 50 sec. The sections were rinsed in distilled water and put in phosphotungstic acid and phosphomolybdic acid for 10 min. Afterwards, they were stained with Aniline blue for 4 min. After covered with coverslips and sealed, the sections were photographed using an Olympus BX53F microscope.

### Statistical analysis

Data were analyzed via two-way ANOVA unless stated, and differences were considered statistically significant at *p* < 0.05.

## Results

### Expression and phosphorylation of AS160/TBC1D4 in mouse heart

**Fig 1:**
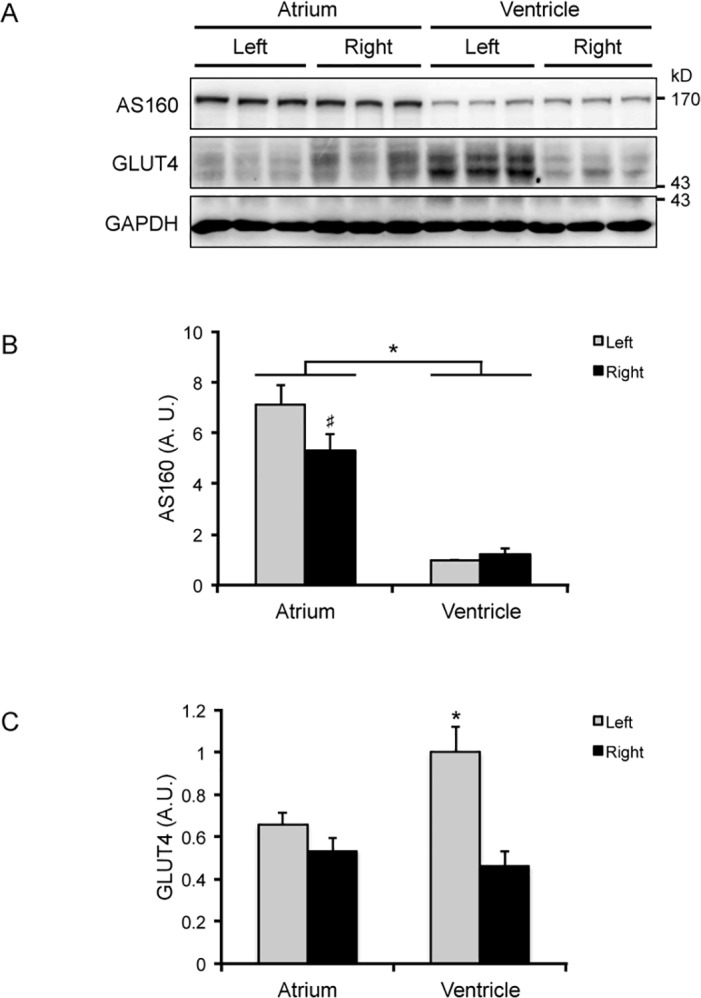
The expression of AS160/TBC1D4 and GLUT4 in the four chambers of mouse heart. A, Immunoblotting analyses of AS160/TBC1D4 and GLUT4 in the lysates (40 μg) of the four chambers of mouse heart. GAPDH was used as a loading control. B, Quantitation of AS160/TBC1D4 expression in the four chambers of mouse heart. n = 6. Asterisk indicates *p* < 0.05 (atrium versus ventricle). ♯ indicates *p* < 0.05 (right atrium versus left atrium). C, Quantitation of GLUT4 expression in the four chambers of mouse heart. n = 6. Asterisk indicates *p* < 0.05 (left ventricle versus the other three chambers).

We previously reported that AS160/TBC1D4 is ubiquitously expressed in most mouse tissues and its expression level is relatively high in the heart [22]. A more detailed analysis of expression of AS160/TBC1D4 was first carried out in mouse heart (Fig 1). The expression levels of AS160/TBC1D4 were more than 5-times higher in atria than in ventricles, and its highest expression levels were found in the left atrium (Fig 1A and 1B). This expression pattern of AS160/TBC1D4 was distinct from that of GLUT4 that showed the highest expression level in the left ventricle (Fig 1A and 1C).

**Fig 2:**
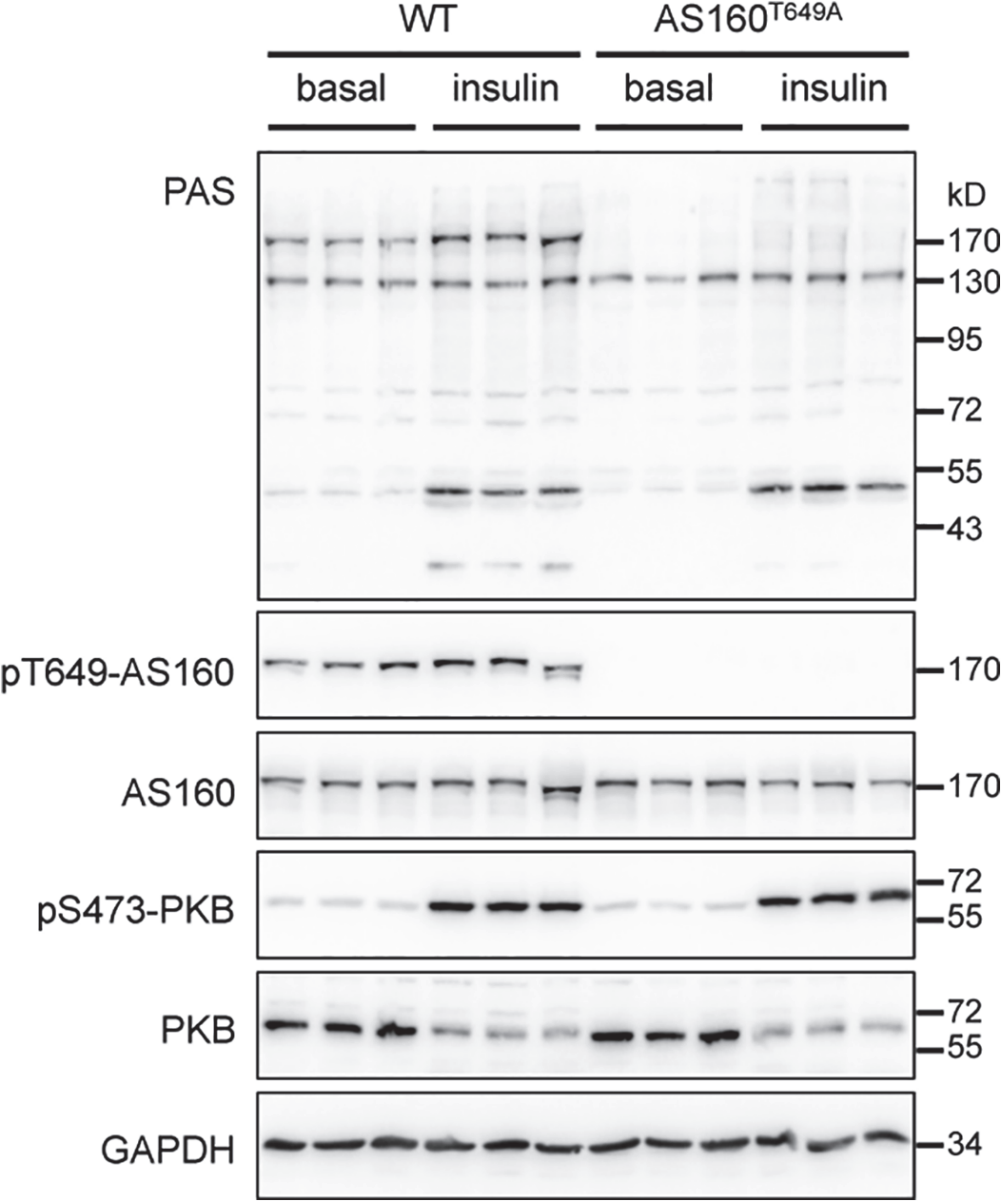
Phosphorylation of AS160/TBC1D4 in mouse heart in response to insulin. PAS-binding signals, phosphorylation of AS160-Thr^649^ and PKB-Ser^473^, and total AS160 and PKB were detected in the lysates (40 μg) of hearts from the wild-type and AS160^Thr649Ala^ knockin mice that were intraperitoneally injected with PBS (basal) or insulin (150 mU per kg of body weight) for 20 min. GAPDH was used as a loading control.

AS160/TBC1D4 was initially identified as a PAS-binding protein in the adipose tissue [10], and the PAS binding site is the phospho-Thr^649^ [18]. We found that AS160/TBC1D4 also represented one of the major PAS-binding phospho-proteins upon insulin stimulation in the heart from the wild-type mice (Fig 2). These PAS-binding signals ~160 kDa were diminished in the heart from the AS160^Thr649Ala^ knockin mice in parallel with undetectable phospho-Thr^649^ signals (Fig 2). The activation of PKB upon insulin stimulation was normal in the heart from these AS160^Thr649Ala^ knockin mice (Fig 2).

### The R-wave amplitude was increased in the hearts from the AS160^Thr649Ala^ knockin mice

We previously reported that the ratio of heart to body weight was increased in the AS160^Thr649Ala^ mice [19]. We next studied the heart’s electrical conduction system of the AS160^Thr649Ala^ mice via electrocardiography (ECG). Interestingly, the R-wave amplitude in the ECG was moderately increased in the heart of the young male AS160^Thr649Ala^ mice (2 month-old) and significantly increased in the heart of the old male AS160^Thr649Ala^ mice (8 month-old) under ambient conditions (Table 1). The rest of ECG parameters appeared normal in the heart of both the young and old male AS160^Thr649Ala^ mice except that the QT interval and QTc were significantly increased in the heart of the old male AS160^Thr649Ala^ mice (Table 1). In the female AS160^Thr649Ala^ mice (2.5 month-old), the amplitudes of the Q-, R- and S-waves were significantly altered while the other ECG parameters remained normal (Table 1). The heart rates were comparable between the wild-type and knockin mice in both genders under these conditions (Table 1).

**Table 1:** Electrocardiographic parameters of the wild-type and AS160^Thr649Ala^ knockin hearts.

ECG parameters	Genotype	Young male		Old male		Female	
		WT (n = 14)	AS160^Thr649Ala^ (n = 9)	WT (n = 13)	AS160^Thr649Ala^ (n = 6)	WT (n = 9)	AS160^Thr649Ala^ (n = 11)
Heart rate		461 ± 13	461 ± 20	384 ± 9	436 ± 26	393 ± 11	388 ± 10
RR Interval (ms)		130.1 ± 4.3	132.8 ± 5.4	157.2 ± 3.8	140.3 ± 8.7	153.7 ± 4.4	155.7 ± 3.8
QRS Interval (ms)		11.5 ± 0.3	11.5 ± 0.2	12.0 ± 0.1	12.3 ± 0.2	12.3 ± 0.3	12.5 ± 0.2
QT Interval (ms)		23.2 ± 0.9	22.6 ± 0.7	19.9 ± 0.3	21.2 ± 0.5*	24.1 ± 0.3	23.5 ± 0.7
QTc (ms)		64.9 ± 3.3	62.3 ± 2.7	49.9 ± 1.1	57.1 ± 2.7*	61.6 ± 1.1	59.7 ± 1.9
JT Interval (ms)		11.7 ± 1.0	11.1 ± 0.8	8.3 ± 0.4	8.9 ± 0.5	11.8 ± 0.3	11.0 ± 0.7
Tpeak Tend Interval (ms)		9.5 ± 1.0	8.9 ± 0.8	6.0 ± 0.3	6.6 ± 0.3	9.1 ± 0.3	8.6 ± 0.6
Q Amplitude (mV)		- 0.041 ± 0.005	- 0.037 ± 0.005	- 0.036 ± 0.004	- 0.043 ± 0.005	- 0.011 ± 0.004	- 0.036 ± 0.006*
R Amplitude (mV)		0.656 ± 0.058	0.798 ± 0.076	0.661 ± 0.025	0.884 ± 0.050*	0.549 ± 0.032	0.734 ± 0.063*
S Amplitude (mV)		- 0.279 ± 0.030	- 0.345 ± 0.036	- 0.255 ± 0.040	- 0.363 ± 0.050	- 0.226 ± 0.049	- 0.348 ± 0.042*
ST Height (mV)		0.094 ± 0.009	0.097 ± 0.013	0.060 ± 0.006	0.057 ± 0.006	0.137 ± 0.024	0.106 ± 0.009
T Amplitude (mV)		0.207 ± 0.017	0.208 ± 0.026	0.135 ± 0.012	0.151 ± 0.032	0.230 ± 0.023	0.224 ± 0.015

Electrocardiography was performed on the anaesthetized AS160^Thr649Ala^ knockin mice and wild-type littermate controls. The male mice were at age of 2 months (young male) and 8 months (old male), respectively, while the female mice were at age of 2.5 months. Student’s t-test was performed and asterisk indicates *p* < 0.05.

### Heart functions were normal in the AS160^Thr649Ala^ knockin mice

We next studied the heart functions of the AS160^Thr649Ala^ knockin mice via echocardiography (Echo). The heart functions including ejection fraction (EF) and fractional shortening (FS) were normal in the young male AS160^Thr649Ala^ mice (Table 2) and remained unaffected in the old male AS160^Thr649Ala^ mice (Table 2). The volume of left ventricles was significantly decreased in the heart from the young male AS160^Thr649Ala^ mice but was normal in the heart from the old male AS160^Thr649Ala^ mice (Table 2). No cardiac hypertrophy was observed in the heart of the male AS160^Thr649Ala^ mice (Table 2). Similarly, the female AS160^Thr649Ala^ mice also displayed normal heart functions (Table 2).

**Table 2:** Echocardiographic parameters of the wild-type and AS160^Thr649Ala^ knockin hearts.

Echo parameters	Genotype	Young male		Old male		Female	
		WT (n = 14)	AS160^Thr649Ala^ (n = 9)	WT (n = 13)	AS160^Thr649Ala^ (n = 6)	WT (n = 9)	AS160^Thr649Ala^ (n = 11)
%EF		53.78 ± 1.19	58.13 ± 2.97	45.24 ± 1.02	45.22 ± 2.46	53.52 ± 1.75	52.65 ± 1.75
%FS		27.37 ± 0.74	30.36 ± 1.93	22.26 ± 0.59	22.27 ± 1.38	26.16 ± 1.23	26.47 ± 1.07
LV Vol; d		65.14 ± 2.40	58.33 ± 1.81*	75.85 ± 1.52	75.82 ± 6.65	49.84 ± 1.74	51.36 ± 2.59
LV Vol; s		30.38 ± 1.74	24.58 ± 2.07*	41.05 ± 1.31	42.34 ± 5.34	22.49 ± 1.72	26.04 ± 2.12
LVID; d		3.85 ± 0.06	3.70 ± 0.05	4.16 ± 0.04	4.12 ± 0.16	3.47 ± 0.05	3.51 ± 0.07
LVID; s		2.81 ± 0.07	2.58 ± 0.09	3.20 ± 0.04	3.21 ± 0.18	2.50 ± 0.08	2.69 ± 0.08
LVAW; d		0.73 ± 0.01	0.74 ± 0.01	0.64 ± 0.00	0.64 ± 0.00	0.69 ± 0.01	0.74 ± 0.01*
LVAW; s		1.00 ± 0.01	1.01 ± 0.03	0.85 ± 0.00	0.86 ± 0.00*	0.90 ± 0.02	0.91 ± 0.02
LVPW; d		0.72 ± 0.02	0.72 ± 0.03	0.75 ± 0.02	0.74 ± 0.03	0.66 ± 0.01	0.72 ± 0.02
LVPW; s		1.01 ± 0.02	1.01 ± 0.03	0.95 ± 0.02	0.96 ± 0.04	0.93 ± 0.02	0.93 ± 0.03
LV Mass (AW)		97.79 ± 2.71	91.89 ± 2.80	103.15 ± 1.96	102.02 ± 7.48	78.34 ± 2.08	84.07 ± 2.99
LV Mass (AW) Corrected		78.23 ± 2.17	73.51 ± 2.24	82.52 ± 1.56	81.62 ± 5.99	62.67 ± 1.67	67.26 ± 2.39

Echocardiography was performed on the anaesthetized AS160^Thr649Ala^ knockin mice and wild-type littermate controls. The male mice were at age of 2 months (young male) and 8 months (old male), respectively, while the female mice were at age of 2.5 months. Student’s t-test was performed and asterisk indicates *p* < 0.05.

### Myocardial infarction after coronary artery ligation were comparable in the heart from the wild-type and AS160^Thr649Ala^ knockin mice

**Fig 3:**
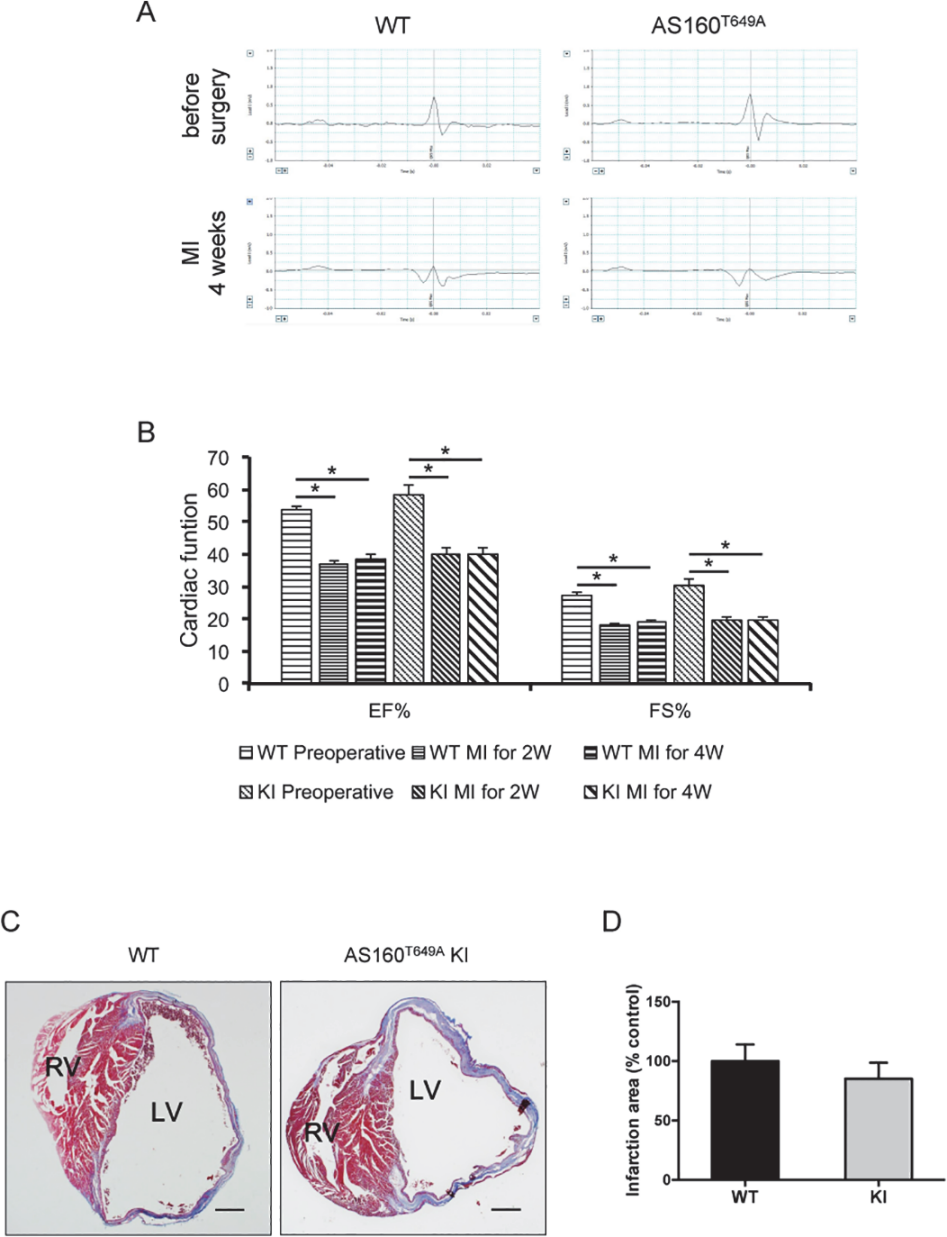
Cardiac functions in the wild-type and AS160^Thr649Ala^ knockin mice before and after myocardial infarction. A, ECG was measured before, and 4 weeks after myocardial infarction in the wild-type and AS160^Thr649Ala^ knockin mice. Representative ECG graphs were shown. B, Cardiac functions including EF and FS were measured via echocardiography before, and 2 and 4 weeks after myocardial infarction in the wild-type and AS160^Thr649Ala^ knockin mice. Asterisk indicates *p* < 0.05. C, Masson’s staining of the infarct heart from the wild-type and AS160^Thr649Ala^ knockin mice 4 weeks after myocardial infarction. D, Quantitation of the infarction areas detected by Masson’s staining. n = 3.

We next induced myocardial infarction in mice via ligation of the left anterior descending coronary artery, and monitored the heart’s electrical conduction system and heart functions of the mice through ECG and Echo before and after surgery. Though the R amplitude in the ECG was modestly larger in the knockin heart than the wild-type heart before surgery (Table 1), the R amplitude was diminished or even inverted in the heart from both genotypes four weeks after surgery, and no obvious difference in the ECG could be observed between the two genotypes after surgery (Fig 3A). The EF and FS were decreased in the wild-type and AS160^Thr649Ala^ mice to similar levels two and four weeks after surgery (Fig 3B). The infarcted regions were also comparable as revealed by Masson’s staining of the infarct heart sections (Fig 3C and 3D).

### TBC1D1 expression was induced in the infarct heart from the wild-type and AS160^Thr649Ala^ knockin mice

**Fig 4:**
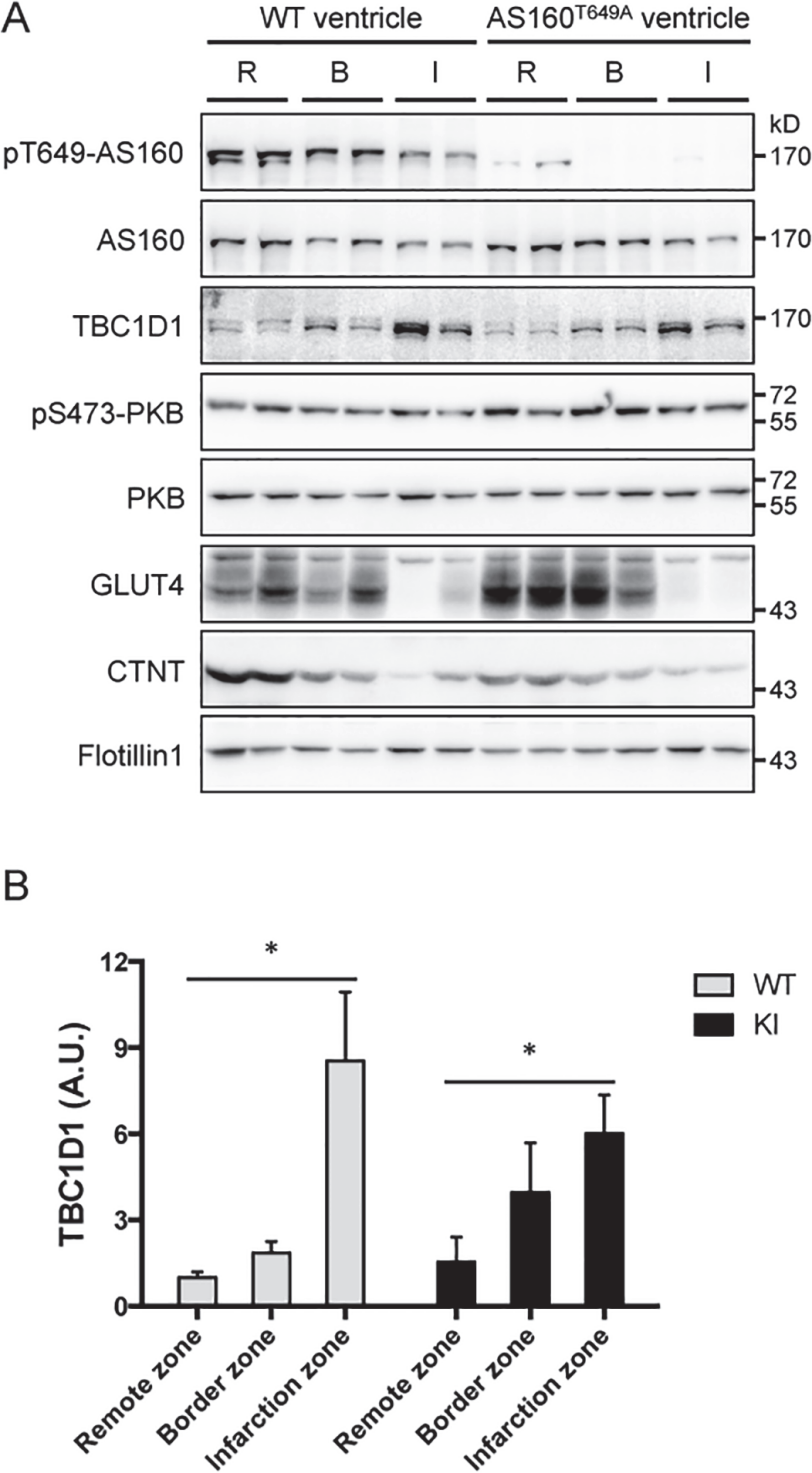
Expression and phosphorylation of AS160 and TBC1D1 in the infarct heart. Immunoblotting analyses of phosphorylation of AS160-Thr^649^ and PKB-Ser^473^, and total AS160, TBC1D1, PKB, GLUT4 and cTnT in the lysates (40 μg) of the infarct zone (I), border zone (B) and remote zone (R) of mouse heart 4 weeks after myocardial infarction. Flotillin-1 was used as a loading control. A, representative blots. B, quantitation of TBC1D1 signals, n = 4. Asterisk indicates *p* < 0.05 (infarct zone versus remote zone).

We investigated the molecular changes in the infarct heart from the AS160^Thr649Ala^ knockin mice and wild-type littermate controls (Fig 4). The expression levels of the cardiac troponin T (cTnT) and GLUT4 were decreased in the border and infarct zones of the heart from both genotypes to similar levels. The expression levels of AS160 were decreased in the infarct zone in parallel with the Thr^649^ phosphorylation of AS160 in the wild-type heart. Similarly, the expression of AS160 was also down-regulated in the infarct zone of the AS160^Thr649Ala^ knockin mice. As expected, the Thr^649^ phosphorylation of AS160 was not detectable in the lysates of the AS160^Thr649Ala^ knockin heart. Interestingly, the expression of TBC1D1 was strongly induced in the infarct zones and also increased in the border zones of the heart from both genotypes (Fig 4A and 4B).

## Discussion

In this study, we found that PKB-mediated phosphorylation of Thr^649^ on AS160/TBC1D4 represented one of the major PAS-binding signals in the heart in response to insulin. Using the AS160^Thr649Ala^ knockin mouse model, we revealed that the Thr^649^ phosphorylation of AS160/TBC1D4 plays an important role in the heart’s electrical conduction system.

The R-wave amplitude was moderately but not statistically increased in the hearts of the young male AS160^Thr649Ala^ knockin mice, and significantly increased in the hearts of the old male and young female AS160^Thr649Ala^ knockin mice under normal conditions. The increased R-wave amplitude is a diagnostic marker for cardiac hypertrophy [23]. Although the ratio of heart to body weight was increased in the AS160^Thr649Ala^ knockin mice, the hearts showed no hypertrophic symptom as evidenced by the heart weight (data not shown) and the left ventricle mass detected by Echo (Table 2). Since the AS160/TBC1D4 regulates trafficking of epithelial sodium channel and Na^+^/K^+^-ATPase in kidney cells [15, 16], it is possible that this RabGAP may regulate ion homeostasis in cardiomyocytes thus affecting the R-wave amplitude. Since the AS160^Thr649Ala^ knockin mutation occurs at the whole-body level, it is also possible that this mutation causes the increased R-wave amplitude indirectly. Apart from the increased R-wave amplitude, the heart of the AS160^Thr649Ala^ knockin mice was normal regarding the heart functions such as the EF and FS. This AS160^Thr649Ala^ knockin mutation also did not severe the heart infarction after artery ligation. It is possible that factors other than Thr^649^ phosphorylation of AS160/TBC1D4 mediate the pathogenesis of dilated cardiomyopathy and cardiac infarction due to the loss of PKB/Akt. For example, other phosphorylation sites on the AS160/TBC1D4 may have a more important role in maintaining heart functions. Another possibility is that other proteins may play a compensatory role for the AS160^Thr649Ala^ knockin mutation in regulating heart functions. In line with this possibility, the expression of the related RabGAP, TBC1D1, was markedly increased in the infarct heart from both AS160^Thr649Ala^ knockin mice and wild-type littermate controls. TBC1D1 and AS160/TBC1D4 display similar GAP activities towards downstream Rabs including Rab2, Rab8a, Rab10 and Rab14 [12]. TBC1D1 can also be phosphorylated by PKB on Thr^590^ that is a paralogous site of Thr^649^ on AS160 [11, 12].

The finding that TBC1D1 is induced in the infarct heart is intriguing and deserved for further investigation in future. The cardiac ischemia due to blockage of the coronary artery causes hypoxia in cardiomyocytes [24]. If the blockage persists, the ischemia will consequently cause cell death of cardiomyocytes but concomitantly trigger fibrosis to maintain the heart functions for a short period. However, this compensatory response may have a deadly consequence that eventually leads to myocardial infarction. It would be interesting in future to find out whether hypoxia or fibrosis in the infarct heart is responsible for the induction of TBC1D1 expression. Another intriguing question is whether the infarction-induced TBC1D1 expression facilitates the decline of heart functions or protects the infarct heart from further damage. Nevertheless, these data demonstrate that TBC1D1 can be used as a novel biomarker to monitor cardiac remodeling during infarction.

In summary, the Thr^649^ phosphorylation of AS160/TBC1D4 does not regulate heart functions under both normal and infarct conditions, but plays an important role in the heart’s electrical conduction system through regulating the R-wave amplitude.
